# Biogenic Elements and Heavy Metals in Hermann’s Tortoises—*Testudo hermanni*: Effect on Serum Biochemistry and Oxidative Status Parameters

**DOI:** 10.3390/ani13132218

**Published:** 2023-07-06

**Authors:** Róbert Kirchner, Soňa Kirchnerová, Filip Tirpák, Marko Halo, Tomáš Slanina, Katarína Tokárová, Anton Kováčik, Michal Miškeje, Veronika Komárňanská, Agnieszka Greń, Grzegorz Formicki, Peter Massányi

**Affiliations:** 1 Institute of Applied Biology, Slovak University of Agriculture in Nitra, Tr. A. Hlinku 2, 949 76 Nitra, Slovakia; robert.kirchner@uniag.sk (R.K.); soni.kirchnerova@gmail.com (S.K.); tomas.slanina@uniag.sk (T.S.); katarina.tokarova@uniag.sk (K.T.); anton.kovacik@uniag.sk (A.K.); peter.massanyi@uniag.sk (P.M.); 2 Institute of Animal Husbandry, Slovak University of Agriculture in Nitra, Tr. A. Hlinku 2, 949 76 Nitra, Slovakia; vevakomar@gmail.com; 3 AgroBioTech Research Centre, Slovak University of Agriculture in Nitra, Tr. A. Hlinku 2, 949 76 Nitra, Slovakia; filip.tirpak@uniag.sk (F.T.); michal.miskeje@gmail.com (M.M.); 4 Institute of Biology, Pedagogical University of Krakow, Podchorazych 2, 30-084 Krakow, Poland; agnieszka.gren@up.krakow.pl (A.G.); grzegorz.formicki@up.krakow.pl (G.F.)

**Keywords:** tortoise, blood biochemistry, oxidative stress, biogenic element, heavy metals

## Abstract

**Simple Summary:**

Animal health is directly linked to viability of the population, which may be affected by anthropogenic activities and diseases. Biomarkers such as serum chemistry and parameters of oxidative balance are good indicators of an overall biological status, providing information on the effects of contaminants on the organism. The objective of this work was to analyze biochemical and molecular indicators and their correlations to biogenic and risk elements in *Testudo hermanni*. Biochemical parameters were analyzed using the commercial kit DiaSys and biochemical analyzer Randox RX Monza. Sodium, potassium, and chlorides were measured using the EasyLite analyzer. Oxidative stress was evaluated using colorimetric and luminometric methods. Quantification of chemical elements in the animal blood was carried out using inductively coupled plasma mass spectrometry. Biochemical values of analyzed samples from Hermann’s tortoises were almost the same as referential values described by multiple authors, with minor aberrations in the total protein parameter. Values of arsenic (As) and nickel (Ni) showed correlation with biochemical parameters and the parameters of oxidative stress. Cadmium (Cd) exhibited correlation with the biochemical parameter aspartate aminotransferase (AST). Finally, this study detected heavy metals and their significant correlations with selected biochemical and molecular parameters in Hermann’s tortoises.

**Abstract:**

Background: Conservation of species diversity is the need of the hour for preserving life forms on Earth. Extinction of any part of the ecosystem has negative impacts on many processes and systems. The objective of this work was to analyze some biochemical and molecular indicators and their correlations to biogenic elements and heavy metals in *Testudo hermanni* (*n* = 16). Methods: Biochemical parameters were analyzed using the commercial kit DiaSys and biochemical analyzer Randox RX Monza. Sodium, potassium, and chlorides were measured using the EasyLite analyzer. Oxidative stress was evaluated using colorimetric and luminometric methods. Quantification of chemical elements in the blood was carried out using inductively coupled plasma mass spectrometry (ICPS). Results: Biochemical values of analyzed samples from Hermann’s tortoises were almost the same as referential values described by multiple authors, with minor aberrations in the total protein parameter. Values of arsenic (As) and nickel (Ni) showed correlation with biochemical parameters and the parameters of oxidative stress. Cadmium (Cd) exhibited correlation with aspartate aminotransferase (AST). Conclusions: This study reports correlations among four heavy metals, and their levels were again correlated with biochemical and molecular parameters in Hermann’s tortoises.

## 1. Introduction

The first fossil record of tortoises dates back 230 million years and, even though they diversified and evolved, they generally stayed constant, and their specific anatomy ensured sufficient evolutional predisposition to survive, even in the times during which the mass extinction of species happened [1]. Urban planning in the last few centuries has led to lowering of numbers of those animals in their natural habitats faster than they can evolve into new species. Today, society is largely focused on streamlining technological procedures, and recent trends have had a largely negative impact on biodiversity, leading to global warming [2]. Animal health is directly linked to the viability of the population, which may be affected by anthropogenic activities and diseases. Biomarkers such as serum chemistry and parameters of oxidative balance are good indicators of an overall biological status, providing information on the effects of contaminants on the organism [3,4].

Pollution caused by heavy metals has the closest correlation with natural (volcanic activity, erosion, and fire caused biological soil weathering) factors and with anthropogenic influences (factories, mining and processing of metals, agriculture) out of all components of the environment [5]. It has been proven that these factors and influences have negative impacts on the quality of the food chain. Higher contamination influences the general health of people and animals [6]. Multiple studies show that heavy metals can build up in fat tissue and consequently disrupt the functions of inner organs or disrupt the nervous or endocrine systems [7,8]. Compounds containing heavy metals are toxic, mutagenic, teratogenic, and carcinogenic for animals in general [9].

There are multiple ways through which heavy metals can enter the organism (consummation, inhalation, through the skin, etc.), thus causing intoxication [10]. Because of this, it is important to monitor the health of animals thoroughly and periodically in polluted areas [6,11]. Cadmium (Cd) is the main pollutant of water-based ecosystems [12]. Studies on the issue of the toxicological potential of Cd have shown that it can increase the levels of reactive forms of oxygen (ROS), leading to much structural and functional damage, such as peroxidation of cellular lipids, destruction of proteins, and DNA mutations [13,14,15,16,17,18]. Influence of heavy metals (mercury—Hg, lead—Pb) has a severe impact on turtles [19,20,21,22]. Anthropogenic influences such as mining, burning of fossil fuels, factory liquidation of waste, usage of agricultural pesticides, and release of waste from water treatment plants may lead to excessive accumulation of trace elements in the environment [23,24,25].

Biochemical analysis can also provide physiological information about a reptile patient’s health status. Many of the biochemistry references available for reptiles are based on study populations of limited size or unknown health status. Because of these limitations, it is best to develop individual patient biochemistry references using known disease-free intervals for the patient (e.g., on annual health checks). These data can then be used in combination with published references to assess a reptile’s health status better [26] and can help with diagnosis of life-threatening conditions and serve as an indicator of problems with important organs of animals.

Tortoises are long-living animals. In the wild they can live up to a few decades, with the largest species living up to hundreds of years [27]. Ex situ management is an important conservation tool that allows the preservation of biological diversity outside natural habitats while supporting survival in the wild. Captive breeding followed by re-introduction is a possible approach for endangered species conservation and preservation of genetic variability [28]. Species and referential intervals are specific to each population and provide the basic information for research.

Oxidation–reduction reactions are an essential part of the metabolism of each cell in the organism. Free radicals that are created as a by-product of those reactions can attack many different biological structures and cause oxidative damage. Oxidative stress is a state during which the creation of free radicals overpowers the antioxidative capacities of the organism. Oxidative damage is the cause as well as the result of many pathophysiological processes, which is what makes the reactions of free radicals with biomolecules a current topic of intensive study and research. Lipids and nucleic acids are biomacromolecules that fall prey to the reactions with free radicals, leading to oxidative damage of their structures [29]. A variety of chemicals may cause oxidative stress because of increased levels of ROS affecting the mitochondrial function, followed by alterations to the enzymatic and/or endogenous antioxidants in blood [30] and other tissues [4,31,32,33,34]. Toxicants are a current dangerous threat, and their concentration determines the toxicity. Increasing levels of environmental pollution lead to larger interest in the interactions of xenobiotics with organic systems. Commonly, blood parameters are used to determine the general health of an organism. The objective of this study was to analyze the influence of selected blood biogenic elements and heavy metals on biochemical and molecular parameters and detection of possible correlations.

## 2. Materials and Methods

### 2.1. Blood Collection and Processing

Tortoises (*n* = 16) were acquired from a private breeder, and blood was collected from the genus *Testudo*, more specifically sp. *Testudo hermanni*, during the reproductive season in June to July. The ages of the analyzed animals ranged from 18 to 25 years and their weight ranged from 780 to 1650 g. There were 7 males and 9 females. Size difference is caused by sexual dimorphism of the species: males grow to about two thirds of the females’ size [35]. The animals were kept in external enclosures. The dimension of the enclosure was 2.5 × 4 m, and it contained a clean drinkable water supply, bushes as the source of shade, and a wooden shed as a hideout. Feeding was secured ad libitum. The main part of the feed consisted of grasses (70%)—*Lolium perenne* and *Festuca*. Herbaceous plants made up about 25% of the tortoise diet—dandelions and plantain. The remaining 5% consisted of leaves of fruit trees, flowers, and ripe fruits. The animals were handled carefully in accordance with the ethical guidelines of the Animal Protection Regulation of the Slovak Republic RD 377/12, complying with the European Union Regulation 2010/63. Experimental protocols were approved by the committee at Slovak University of Agriculture in Nitra, Slovakia.

Blood collection was carried out by trained technicians and a veterinary doctor. Blood was drawn from alive animals from the *sinus subcarapacialis*. In the beginning, the basic steps of the processing and analysis of the specimens were carried out. After specimen collection and transport to the laboratory in thermobox, blood serum was separated for biochemical analysis using centrifugation at 3000 rpm for 10 min [36].

### 2.2. Biochemical Analysis of Blood Serum

Blood serum was analyzed for the purpose of obtaining energy, nitrogen, hepatic and mineral profiles. Analysis was carried out in the laboratory of clinical biochemistry and hematology using the commercial kit DiaSys (Diagnostic system GmbH, Holzheim, Germany) with the Rx Monza instrument (Randox laboratories Ltd., Crumlin, UK). Some of the parameters of the mineral profile (sodium, potassium, and chlorides) were analyzed using the EasyLite Plus machine (The Hague, The Netherlands) [37,38,39].

### 2.3. Total Oxidant Status (TOS)

The principle of TOS analysis is based on the oxidation of ferrous ions-o-dianisidine complexes by the oxidants present in the sample to ferric ions. The process of the oxidation reaction is supported by the glycerol molecules present in the reaction solution. Then, the ferric ions form a colored complex with xylenol orange in the acidic environment of the reaction solution. The color intensity, which can be measured spectrophotometrically, corresponds to the total amount of oxidant molecules present in the sample. The assay was calibrated using hydrogen peroxide, and the results are expressed as μmol H_2_O_2_/L [40]. Briefly, reaction solutions 1 and 2 (TOS R1 and TOS R2) were prepared. The TOS R1 consisted of 150 μmol xylenol orange disodium salt (Sigma-Aldrich, St. Louis, MO, USA), 140 mmol sodium chloride (Sigma-Aldrich, St. Louis, MO, USA), and 1.35 mol glycerol (Centralchem, Bratislava, Slovakia) in 25 mmol H_2_SO_4_ (Sigma-Aldrich, St. Louis, MO, USA). The TOS R2 was composed of 5 mmol ferrous ammonium sulfate hexahydrate (Centralchem, Bratislava, Slovakia), and 10 mmol o-dianisidine dihydrochloride (Sigma-Aldrich, St. Louis, MO, USA) in 25 mmol of sulfuric acid (Sigma-Aldrich, St. Louis, MO, USA). Standards (H_2_O_2_) and the samples’ plasma were transferred in doubles to a 96-well plate in a volume of 35 μL. A reference reading at 560 nm using a Glomax Multi+ Detection System plate reader (Promega, Madison, WI, USA) was realized following the addition of 225 μL TOS R1. After 10 min incubation, 11 μL TOS R2 was added to each well, and the absorbance was spectrophotometrically assessed at the same wavelength after 3 min of incubation [36,40].

### 2.4. Ferric Reducing Ability of Plasma (FRAP)

The FRAP analysis was carried out based on Benzie and Strain [41]. The FRAP reagent contained 10 mmol/L TPTZ (2,4,6-tripyridyl-s-triazine; Sigma-Aldrich, St. Louis, MO, USA) solution in 40 mmol/L HCl (Centralchem, Bratislava, Slovakia), 5 mL of 20 mmol/L ferric chloride (Centralchem, Bratislava, Slovakia), and 50 mL of 0.3 mmol/L acetate buffer (pH = 3.6; Centralchem, Bratislava, Slovakia). Samples (100 μL) were mixed with a 3 mL FRAP reagent, and the absorbance of the reaction mixture was evaluated at 593 nm using a Multiskan FC spectrophotometer (ThermoFisher Scientific, Vantaa, Finland). The absorbance reading was repeated after 4 min of co-incubation of samples/standards with the reaction solution. The final value for each sample was calculated using the FRAP equation ΔA = M2 − M1 [36,40].

### 2.5. Superoxide Dismutase (SOD)

SOD was analyzed on the Randox RX Monza (Randox Laboratories, Crumlin, UK) using a RANDOX assay kit RANSOD (Randox Laboratories, Crumlin, UK) following the manufacturer’s instructions. SOD was spectrophotometrically quantified at 505 nm using a Multiskan FC (ThermoFisher Scientific, Finland) and expressed as U/mg of total protein [36].

### 2.6. Glutathione Peroxidase (GPx)

Activity of GPx was analyzed using the commercially available kit RANSEL (Randox Laboratories, Crumlin, UK) and Randox RX Monza analyzer (Randox Laboratories, Crumlin, UK). The decrease in absorbance was measured at 340 nm. Enzyme activity was expressed as U/mg of total protein. The measurement of GPx was based on catalyzation of glutathione with cumene hydroperoxide. With glutathione reductase and nicotinamide adenine dinucleotide phosphate present, the oxidated glutathione immediately changed into its reduced form with the oxidation of NADPH into NADP+. The decrease in absorbance was evaluated [42,43].

### 2.7. Total Antioxidant Status (TAS)

The assessment of TAS originates from the ability of all antioxidants in the sample to neutralize a prooxidant compound. The TAS Randox (Randox Laboratories, Crumlin, UK) assay follows an incubation of ABTS (2,2′-Azino-di-[3-ethylbenzthiazoline sulphonate]) with a peroxidase (metmyoglobin) and H_2_O_2_ to produce the ABTS+ radical. This has a relatively stable blue–green color, which may be measured at 600 nm. Antioxidants present in the sample suppress this color production to a degree, which is proportional to their concentration. TAS was assessed using the Genesys 10 spectrophotometer (Thermo Fisher Scientific Inc., Waltham, MA, USA) and was expressed as μmol/mg protein [44,45].

### 2.8. Detection of Essential and Heavy Metals in Blood Serum

All chemicals used in the process of preparing the sample were clean. Samples (0.5 mL) were split in the Ethos UP high-performance microwave digestion system (Milestone. Srl, Sorisole, BG, Italy) in 5 mL HNO_3_ solution (TraceSELECT^®^, Honeywell Fluka, Morris Plains, NJ, USA) and 1 mL of H_2_O_2_ (30% for trace analysis, Merck, Darmstadt, Germany). All samples along with a blind sample were mineralized in accordance with the recommendations of the manufacturer. The method uses warming and cooling steps. During the heating phase, all the samples were continually warmed up to 200 °C for a duration of 15 min, the temperature was kept the same for another 15 min, and during the next 15 min the samples were actively cooled down so the temperature dropped to 50 °C. The samples were then filtered using Sartorius filtration discs (class 390) (Sartorius AG, Goettingen, Germany) into a volumetric flask and then filled with ultraclean water until the solution reached 50 mL or in case of the need for further diluting. Dilution was considered within the final collection of results. Quantification of chemical elements (Ag, As, Al, Ba, Ca, Cd, Co, Cr, Cu, Fe, Li, Mg, Mn, Mo, Ni, Pb, Sb, Se, Sr, and Zn) present in blood serum was carried out using inductively coupled plasma emission spectrophotometer (ICP Thermo iCAP 7000 Dual, Thermofisher Scientific, Waltham, MA, USA). Multielement standard solution V for ICP (Sigma-Aldrich Production GmbH, Buchs, Switzerland) was used for calibration [36]. Limits of quantification (LOQs) for all evaluated elements are summarized in Table 1.

### 2.9. Statistical Analysis 

For the statistical processing of the results, the statistical program GraphPad Prism 6 (GraphPad Software, Inc., La Jolla, CA, USA) was used along with paired student t-tests. Determination of correlations between each element in blood serum and biochemical parameters, respectively, between evaluated elements and oxidative stress was analyzed using the standard Pearson’s parametrical test of correlations, based on Gauss distribution of population. Levels of dependencies were calculated as follows: 0–0.33 (weak correlation), 0.33–0.66 (medium correlation), and 0.66–1 (strong correlation). The provability of dependencies/correlations was processed into 3 levels of significance: *p* < 0.05, *p* < 0.01, and *p* < 0.001. Based on the final results, heatmaps were created describing the correlation between individual indicators [36].

## 3. Results

The blood serum of tortoises (*n* = 16) was analyzed on three levels—biochemical profile, oxidative status, and the appearance of chemical elements. After the primary analysis, the results of each one was statistically evaluated using correlations between the parameters, and they were transcribed into a visual depiction in the form of heatmaps.

### 3.1. Concentrations of Biochemical Parameters in Blood Serum

Concentrations of biochemical parameters in blood serum are shown in Table 2. The concentration of proteins was 54.91 g/L. In the mineral profile of blood serum, the concentrations of P, Ca, Na, K, and Mg were evaluated. The lipid profile of blood plasma was monitored using cholesterol (9.94 mmol/L) and TAG (2.27 mmol/L).

### 3.2. Parameters of Oxidative Stress in Blood Serum

The oxidative status in the blood serum of tortoises was monitored using the values of TOS (4.96 μmol H_2_O_2_/g TP) and FRAP (297.7 μmol Fe^2+^/g TP). The TAS level was 32.29 mmol/L TP (Table 3).

### 3.3. Chemical Composition of Blood Serum

The results of the concentration of chemical elements recorded in the blood serum of tortoises were: As 139.1 µg/L, Cd 98.20 µg/L, and Ni 237.7 µg/L. Further results are recorded in Table 4. Concentrations of Al, Co, Cr, Hg, Mo, Pb, Sb, and Se were under the level of quantification.

### 3.4. Correlation Analyses

**Figure 1:**
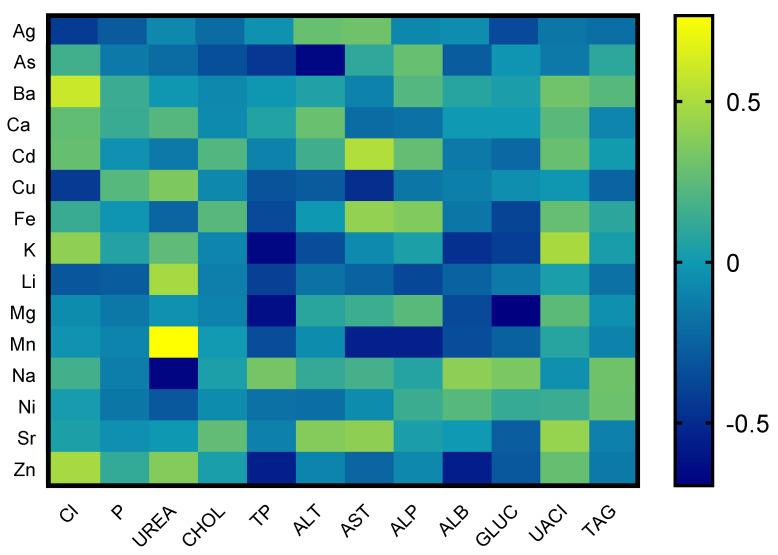
Correlations between chosen biochemical parameters and chemical elements in tortoise.

Correlations between the elements and the results of the biochemical parameters are listed in Figure 1. Correlation analysis of chemical elements and biochemical parameters proved strong between the concentrations of biochemical parameters and chemical elements—heavy metals. The correlation of As with alanine transferase (ALT) showed a strong negative correlation, whereas Cd with aspartate transferase (AST) showed a positive correlation. The correlation of Cu with chlorides (Cl^l−^) and AST showed a strong negative correlation. Total proteins showed negative correlations with Mg, K, and Zn. Urea showed a positive correlation with Mn and a negative correlation with Na, and glucose had a strong negative correlation with Mg. Mn showed a strong negative correlation with AST and ALP, and albumin showed a strong negative correlation with Zn. The relationship of chlorides (Cl^l−^) and barium (Ba) also showed a strong positive correlation. The rest of the evaluated biochemical parameters showed low or medium interactions with chemical elements.

**Figure 2:**
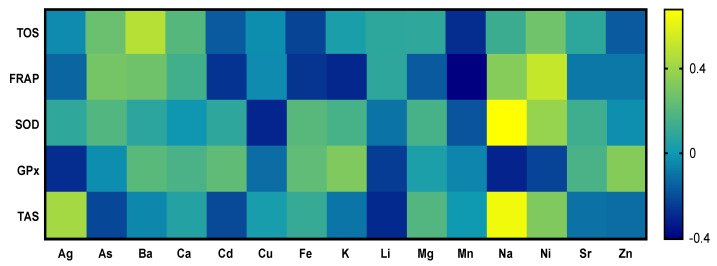
Correlations between chosen parameters of oxidative stress and chemical elements of tortoises.

In Figure 2, correlations between chemical elements and the results from parameters of oxidative stress are depicted. Correlation analysis of chemical elements and parameters of oxidative stress showed a positive correlation between nickel and the ability of plasma to reduce iron. The parameters of oxidative stress, TAS and SOD, showed a strong positive correlation with sodium. The parameter of oxidative stress, FRAP, showed a strong negative correlation with cadmium, potassium, and manganese. The parameter of oxidative stress, GPx, showed a strong negative correlation with sodium and aluminum. Other evaluated metals and biogenic elements showed low or medium interactions with parameters of oxidative stress.

## 4. Discussion

Blood collection and analysis are one of the most common and accessible diagnostic procedures for captive tortoise species. According to Dickinson et al. [46], knowledge of biochemical parameters of tortoises kept in captivity and the ones living freely in the wild is important for the evaluation and further conservation strategy of the species. Blood parameters can diagnose tortoise diseases, and more importantly they can help the physiological status of the population [47]. Ex situ strategies, such as captive breeding and re-introduction, have become an important conservation tool used to combat biodiversity loss by recovering locally extinct populations [28,48,49], and these strategies also include diagnostic methods at various levels.

Since *T. hermanni* is an endothermic species, it can be affected by external conditions and outer and inner factors that affect its blood parameters. Studies on biochemistry are restricted in the *Testudo* genus. Hamooda et al. [50] determined the sexual and seasonally associated changes in biochemical parameters of *T. hermanni*. Nieto-Claudin et al. [51] reported that referential intervals of tortoises kept in human care are more and more available and have a big value. This agrees with the present study, and our results may be used in further studies and evaluations with the goal of more comparative studies, including differences in sex and seasonality.

The results of our study correspond with the findings of multiple authors indicating referential values of the *Testudo* genus and *T. hermanni*. The exception was mostly made for total proteins. Chitty and Raftery [52] stated reference values of total proteins as 23.0–43.0 g/L. Similar findings of total proteins were reported in another study [53]. Knotek et al. [54] noted the highest concentration of total proteins at 75 g/L. It is possible to agree with those statements, since our highest levels, measured in pregnant females before the second laying, were 90 g/L. This was probably caused by stress due to handling before laying. The level of the values outside the laying period were up to 60 g/L. Normal levels of total proteins in reptiles are lower than that of mammals [55]. Andreani et al. [55] stated that the whole genus *Testudinata* has generally lower levels of albumins and higher levels of globulins, and an overall lower A/G ratio, which was also proved by our analysis. 

Diagnostic methods often contain information about physical diagnosis, along with morphological and biochemical analysis, which provide important information about the health of the animal or the health of the whole population [56,57,58]. Important aspects focused on the difficulties with descriptions of referential intervals of all wild animals using data from taxonomically related species seem helpful only in certain situations. Conclusions should be drawn with utmost care and consideration of large interspecific differences. Today, unlike avian and mammalian species, there is a problem with evaluating the general health status of reptiles. Evaluation of such studies, particularly the results, are important for further research and study [59].

The threats that endanger animals in the wild today are the loss and fragmentation of their biotope, illegal trade, introduced and invasive species, global warming, destruction/consumption of their eggs by introduced predators, illnesses, trauma, or antimicrobial resistance, although negative effects of toxicants pose a threat to life and health of animals and humans, which must also be considered [51,60,61,62].

Data on the influence of toxicants on sea turtles are well processed and we know that, in sea turtles, high levels of Cd are detected [63,64], although research on the effects of Cd on freshwater turtles or tortoises is still not sufficient. Cd is a heavy metal, mostly spread in freshwater ecosystems [65,66,67], because of the anthropogenic influence of mining and coal combustion [68]. In their study, Huo et al. [18] found that the activity of ALT and AST in blood plasma have a common correlative relationship with Cd in the blood of *Mauremys reevesii* species. Those findings are partially identical with our results. The activity of AST had a positive correlation with the amount of Cd found in *T. hermanni* blood; on the other hand, ALT did not have a provable mutual correlation in our samples. One of the interesting things is that the activity of ALT had a strong negative correlation with arsenic. Those differences could be caused by the specifics of each species. This heavy metal has no known biological function and is increasing in the atmosphere due to mining, smelting, refining of ores, burning of fossil fuels, and waste incineration [69]. Cadmium exposure has shown to result in oxidative stress, damage to DNA and RNA, disruption in bone formation, and toxicity to the kidney and liver, the target organs [70,71], yet, information on the effects of cadmium on the health and immune function of *T. hermanni* are lacking. The results of our study show that heavy metals and biogenic elements have a mutual correlative relationship with the parameters of oxidative stress (SOD, FRAP, TOS, GPx). Those results are the same as the results of a study by Huo et al. [16], in which they suggest that Cd is the cause of oxidative stress in turtles. Correlative relationships of oxidative stress and Cd are also suggested by Bonsignore et al. [72] in their study in which they characterized the time dependence between the accumulation of metals and oxidative stress in tissues of *Sparus aurata*, which stimulated biochemical paths to develop into a state that would affect the health of the animal. Those claims are supported by our study. Heavy metals have a negative influence and there are mutual correlations between Cd and the individual parameters of oxidative stress as well as biochemical parameters.

Most of the studies dealing with the issue of heavy metals and their impact on the health status of turtles and tortoises follow wild populations of animals. It is important to note that there are many complex factors and relationships that occur at the transcriptional level [73]. However, there are no toxicity threshold values for metals in turtles and tortoises, and non-essential elements such as Al, As, Cd, Cr, Hg, and Pb are toxic even at lower concentrations in different organisms [74]. Iron is important in processes such as blood production, DNA synthesis, cellular respiration, and cell growth, as well as proliferation [73,75,76]. On the contrary, Pietrangelo [77] reported that high levels of iron can exchange electrons with a wide variety of substrates that may also lead to the formation of ROS. This phenomenon causes damage to cells and tissues by attacking DNA, proteins, and membrane lipids [78].

Pinya et al. [79] stated the levels of superoxide dismutase and glutathione peroxidase in the blood of sea turtles, *Caretta caretta*. Their SOD results in the PBMCs (peripheral blood mononuclear cells) were 1.54 (pKat/mg prot) and GPx 6.98 (nKat/mg prot), and SOD in the blood plasma was 11.8 (pKat/mg prot). The differences in analysis could be caused by different methods as well as special differences. Zhang et al. [80] stated the activity of SOD and GPx in the brain, liver, and kidneys of the Chinese Soft-Shelled Turtle (*Pelodiscus sinensis*). High SOD activity was detected in the liver and kidneys and the highest GPx activity was detected in the liver. Our results of SOD were detected at a lower level in blood serum than SOD values in tissue of internal organs. With the other evaluated parameters, TOS and FRAP, which show us the cell reaction to oxidative stress, there is a significant absence of basic knowledge. On the topic of TOS, there is no other study, so our study provides the first information on the parameter in tortoises of any species. For the parameter FRAP, there has only been one study published, which was carried out on the *Chinemys reevsesii* species of turtle. Islam et al. [81] evaluated FRAP in muscle cells after killing the animals. The final values of FRAP were only shown in figures of evaluated absorbance (A_700nm_ = 0.300) in comparison with our results (297.70 µmoL Fe^2+^/g TP), and because of this it is impossible to compare these studies.

A limitation of our study was the number of animals. For this reason, we did not divide the animals based on sex or age (18–25 years). These factors, as have already been pointed out in other animal species, have an impact on the bioaccumulation of heavy metals in a given organism. In the future, it would be appropriate to focus on these factors as well and to confirm whether this fact also applies to tortoises.

## 5. Conclusions

Blood profile studies in captive reptiles are being carried out for scientific, educational, or commercial reasons. Blood analysis is a relatively noninvasive method than can provide important clinical information about the health and physiological condition of animals. In surroundings with high concentrations of heavy metals, this contamination is several times higher, which poses an extreme health risk for each population. Cd has a high acute toxicity as proven by tests carried out over a short period of time. In fact, high exposure to this element over a short period of time can have a negative effect on the health of animals. It mostly binds to the liver and affects the metabolism of saccharides. In the present study, we report a significant correlation between Cd and the biochemical parameter AST. In fact, four biogenic heavy metals (As, Cd, Ni, Zn) and their significant correlations with chosen biochemical and molecular parameters are noted. However, further studies in tortoises are mandatory to understand how these contaminants can influence the fitness and future of endangered populations.

## Figures and Tables

**Table 1:** The LOQs for each monitored chemical element.

Chemical Element	LOQs (µg·L)
Ag	0.3
Al	0.2
As	1.5
Ba	0.03
Ca	0.01
Cd	0.05
Co	0.15
Cu	0.3
Fe	0.1
Li	0.06
Mg	0.01
Mn	0.03
Mo	0.5
Ni	0.3
Pb	0.8
Sb	2.0
Se	2.0
Sr	0.01
Zn	0.2

**Table 2:** Biochemical profile—tortoises (*n* = 16).

Parameter	Average	SD	Minimum	Maximum
Na (mmol/L)	130.40	8.00	118.10	142.40
K (mmol/L)	5.47	0.88	4.27	7.48
Cl (mmol/L)	107.10	7.60	97.2	124.50
P (mmol/L)	1.98	2.04	0.91	9.50
Ca (mmol/L)	3.03	0.90	1.18	4.59
Mg (mmol/L)	1.93	0.72	0.40	3.59
Urea (mmol/L)	3.44	4.85	0.16	17.99
Cholesterol (mmol/L)	6.94	3.78	2.50	17.01
Total proteins (g/L)	54.91	24.12	16.02	96.84
Albumin (g/dL)	2.13	0.90	0.68	4.10
ALT (µkat/L)	0.14	0.05	0.05	0.21
AST (µkat/L)	1.32	0.54	0.02	2.37
ALP (µkat/L)	19.84	7.37	7.55	35.51
Glucose (mmol/L)	3.14	0.44	2.37	3.99
Uric acid (mg/dL)	6.75	4.86	0.67	16.71
Triacylglyceride (mmol/L)	2.27	3.05	0.11	11.54

**Table 3:** Concentration of oxidative stress parameters—tortoises (*n* = 16).

Parameter	Average	SD	Minimum	Maximum
TOS (µmoL H_2_O_2_/g TP)	4.96	2.05	2.96	9.82
FRAP (µmoL Fe^2+^/g TP)	297.70	159.00	134.10	602.40
SOD (U/mL TP)	1.33	0.88	0.00	3.20
GPx (U/L TP)	32.29	13.92	14.56	72.98
TAS (mmol/L TP)	0.49	0.27	0.00	1.14

**Table 4:** Concentration of chemical elements—tortoises (*n* = 16).

Parameter	Average	SD	Minimum	Maximum
Ag (µg/L)	9.31	10.97	0.30	38.60
As (µg/L)	139.10	310.60	1.50	1026.00
Ba (µg/L)	54.60	55.35	0.60	164.00
Cd (µg/L)	98.20	63.77	0.05	199.90
Cu (µg/L)	1322.00	579.40	834.60	2813.00
Fe (mg/L)	382.10	116.40	196.90	577.80
Li (µg/L)	13.62	9.14	3.60	34.60
Mn (µg/L)	54.04	21.22	33.50	120.90
Ni (µg/L)	237.70	389.20	0.30	1490.00
Sr (µg/L)	167.70	78.88	35.50	298.60
Zn (µg/L)	6447.00	1552.00	3995.00	8804.00

## Data Availability

Not applicable.
